# Clinical differences between adipose‐ and muscle‐layer cesarean scar endometriosis: A retrospective observational study

**DOI:** 10.1002/ijgo.70399

**Published:** 2025-07-21

**Authors:** Emre Erdem Taş, Mohammad İbrahim Halilzade, Melike Doğanay, Ayse Filiz Yavuz

**Affiliations:** ^1^ Gynecology and Obstetrics Department University of Ankara Yıldırım Beyazıt Ankara Türkiye; ^2^ Gynecology and Obstetrics Department University of Health Sciences Ankara City Hospital Ankara Türkiye

**Keywords:** abdominal wall, CA125 antigen, cesarean section, endometriosis

## Abstract

**Background/aim:**

This study evaluated cesarean section scar endometriosis (CSSE) characteristics and compared adipose‐ and muscle‐layer CSSEs.

**Materials and methods:**

We analyzed 53 patients who underwent CSSE surgery between 2019‐2024, with 38 (71.7%) having adipose‐layer and 15 (28.3%) having muscle‐layer CSSE.

**Results:**

Patients’ mean age was 33.4 ± 5.9 years. Symptoms began 40.4 ± 23.5 months after cesarean section. CSSE occurred at scar corners in 42 patients (79.2%) and midline in 11 (20.8%). Mean preoperative CA‐125 was 24.1 ± 14.8 U/mL. Groups differed in symptoms, location, and CA‐125 levels. Adipose‐layer CSSE commonly presented with swelling at corners, while muscle‐layer CSSE showed pain at midline. Muscle‐layer CSSE had higher CA‐125 levels (38.2 ± 19.2 vs. 18.5 ± 7.3 U/mL; *P* = 0.01). The CA‐125 cutoff between groups was 30 U/mL. Pain (odds ratio [OR], 7.2; *P* = 0.03) and elevated CA‐125 (≥30 U/mL) (OR, 19.6; *P* < 0.01) independently associated with muscle‐layer CSSE.

**Conclusion:**

These findings may have important implications in clinical practice, potentially aiding in the localization of undefined lesions, surgical planning, and monitoring of recurrence risk.

## 1 INTRODUCTION

Endometriosis is a benign gynecologic disease characterized by the presence of sex hormone‐dependent endometrial glands and stroma located outside the uterine cavity.^1^ The lesions are typically located on the surface of the pelvic peritoneum and ovaries but may occur outside the abdominal cavity.^1^, ^2^ The presence of endometriosis on the abdominal wall, extending from the peritoneal layer to the skin, is defined as abdominal wall endometriosis (AWE). This condition typically occurs in women of reproductive age and is often associated with previous abdominal surgeries, particularly cesarean sections.^3^, ^4^, ^5^

Cesarean section scar endometriosis (CSSE), which refers to AWE associated with previous cesarean sections, accounts for over half of all AWE cases, with an incidence of 0.03%–0.45%.^4^, ^6^, ^7^ CSSE commonly presents with complaints of pain or swelling around the cesarean scar line a few years after the surgery.^3^, ^4^, ^6^, ^7^, ^8^, ^9^, ^10^, ^11^, ^12^ The diagnosis is based on clinical suspicion and imaging studies, particularly ultrasonography.^6^ Additionally, the role of serum cancer antigens, such as cancer antigen 125 (CA125), in the diagnosis and management of CSSE remains unclear.^7^, ^13^ Surgical removal of lesions relieves symptoms with low recurrence rates.^4^, ^7^, ^13^ However, our knowledge of CSSE remains limited because of the lack of case reports and observational studies. The etiology, risk factors, and clinical and laboratory characteristics of CSSE have not yet been elucidated.

CSSE usually occurs within the adipose or muscle layers of the abdominal wall.^3^, ^11^, ^14^ Previous studies have assessed the clinical characteristics of patients with CSSE without classifying them based on their location (i.e. adipose‐ and muscle‐layer CSSE). In this study, we assessed the clinical characteristics of patients with CSSE treated at our hospital and compared adipose‐ and muscle‐layer CSSE based on the examined parameters.

## 2 MATERIALS AND METHODS

In this retrospective observational cohort study, we reviewed the medical records of 57 patients who underwent surgery for suspected CSSE at a tertiary referral clinic in Ankara, Turkey, between June 2019 and June 2024. The preliminary diagnosis was based on the patient's symptoms, clinical suspicion, and abdominal ultrasonographic findings suggestive of endometriosis. The Ankara City Hospital Clinical Research and Ethics Committee (approval no.: E.1–24‐832) approved this study, and all participants provided oral informed consent before their inclusion. This study was conducted in accordance with the 1964 Declaration of Helsinki and its later amendments, and was reported in accordance with the Strengthening the Reporting of Observational Studies in Epidemiology (STROBE) statement.

Data regarding patient demographic characteristics (i.e. age and parity), medical and surgical history, time interval between the last cesarean section and the initiation of symptoms (months), main symptoms (i.e. pain or swelling around the cesarean section scar), transvaginal ultrasound findings, maximum diameter of the endometriotic mass (mm) on abdominal ultrasound, preoperative CA125 levels, surgical details concerning the CSSE position (i.e. location [at the left or right corner or midline] and depth [within the adipose or muscle layers of the abdominal wall]), and histopathologic examination results were obtained from the hospital's medical database. Patients with histopathologically confirmed benign or malignant neoplasms or chronic granulomatous reactions were excluded from this study.

In our clinical practice, the preoperative surgical readiness of patients with CSSE (i.e. physical examination, serum CA125 levels, and ultrasonographic examinations) was assessed during the mid‐luteal phase, and the surgical procedures were performed in the early follicular phase of the next menstrual cycle. We believe that this surgical approach helps to better differentiate endometriotic lesions from the surrounding intact tissues during surgery.

This study aimed to identify the clinical characteristics of patients with CSSE. Patients were divided into two groups based on the location of the CSSE: lesions within the adipose layer of the abdominal wall, regardless of their proximity to the rectus sheath, were grouped as adipose‐layer CSSE, whereas lesions within the muscle layer were grouped as muscle‐layer CSSE. Groups were compared based on the investigated parameters.

Descriptive parameters were expressed as mean ± standard deviation for continuous variables and as numbers and percentages for categorical variables. The groups (adipose‐layer CSSE vs. muscle‐layer CSSE) were compared using independent sample *t* tests and *χ* ^2^ tests. Receiver operating characteristic curve analysis was used to determine the cut‐off value of serum CA125 levels to discriminate between the adipose‐ and muscle‐layer CSSE groups. Variables with *P* values less than 0.05, including main symptoms, CSSE location, and serum CA125 levels, were included in the binary logistic regression analysis to identify the independent factors associated with muscle‐layer CSSE. Statistical analyses were performed using the Statistical Package for the Social Sciences (SPSS) for Windows (version 21.0; IBM Corp., Armonk, NY, USA). Odds ratios (ORs) and 95.0% confidence intervals (CIs) were calculated, with statistical significance set at a *P* value less than 0.05.

## 3 RESULTS

**FIGURE 1:**
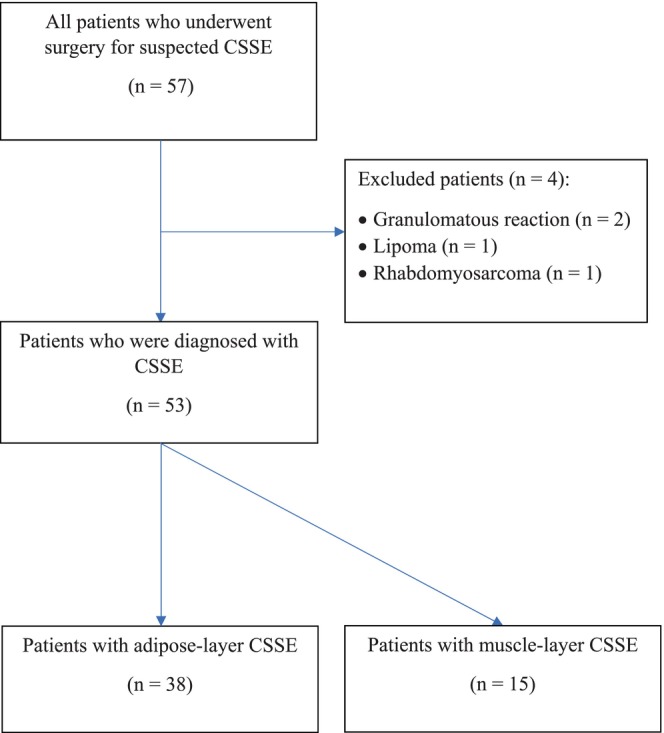
Flow chart of the study population. CSSE, cesarean section scar endometriosis.

After excluding four patients whose histopathologic examinations revealed granulomatous reactions, lipoma, and rhabdomyosarcoma, a total of 53 patients (92.9%) were included in the study. Among them, 38 patients (71.7%) had adipose‐layer CSSE and 15 patients (28.3%) had muscle‐layer CSSE. The flowchart of the study population is shown in Figure 1.

All the patients were of reproductive age and the mean age and parity were 33.4 ± 5.9 years and 2.0 ± 0.8, respectively. Of the eight patients (15.1%) with concurrent medical conditions, five (9.4%) had hypothyroidism, two (3.8%) had multiple sclerosis, and one (1.9%) had type 2 diabetes mellitus. Twenty‐six patients (49.1%) had only one previous cesarean section, whereas 27 patients (51.9%) had two or more previous cesarean sections. Additionally, one patient (1.9%) had a history of laparoscopic ovarian endometrioma cyst excision before the last cesarean section.

The mean time interval from the last cesarean section to the initiation of symptoms was 40.4 ± 23.5 months. Of the 53 patients, the major symptom was pain in 23 (43.4%) and 30 (56.6%) had swelling. Transvaginal ultrasonography revealed no endometriomas or similar cystic structures in the ovaries of any of the patients. The mean diameter of the CSSE on abdominal ultrasonography was 25.0 ± 12.6 mm, and the mean preoperative serum CA125 level was 24.1 ± 14.8 U/mL. Serum CA125 levels were 35 U/mL or greater in 11 of the 53 patients (20.7%). CSSE was located at the corners of the cesarean section scar in 42 patients (79.2%) and at the midline in 11 patients (20.8%). In contrast, CSSE was located within the adipose and muscle layers in 38 (71.7%) and 15 (28.3%) patients, respectively.

**FIGURE 2:**
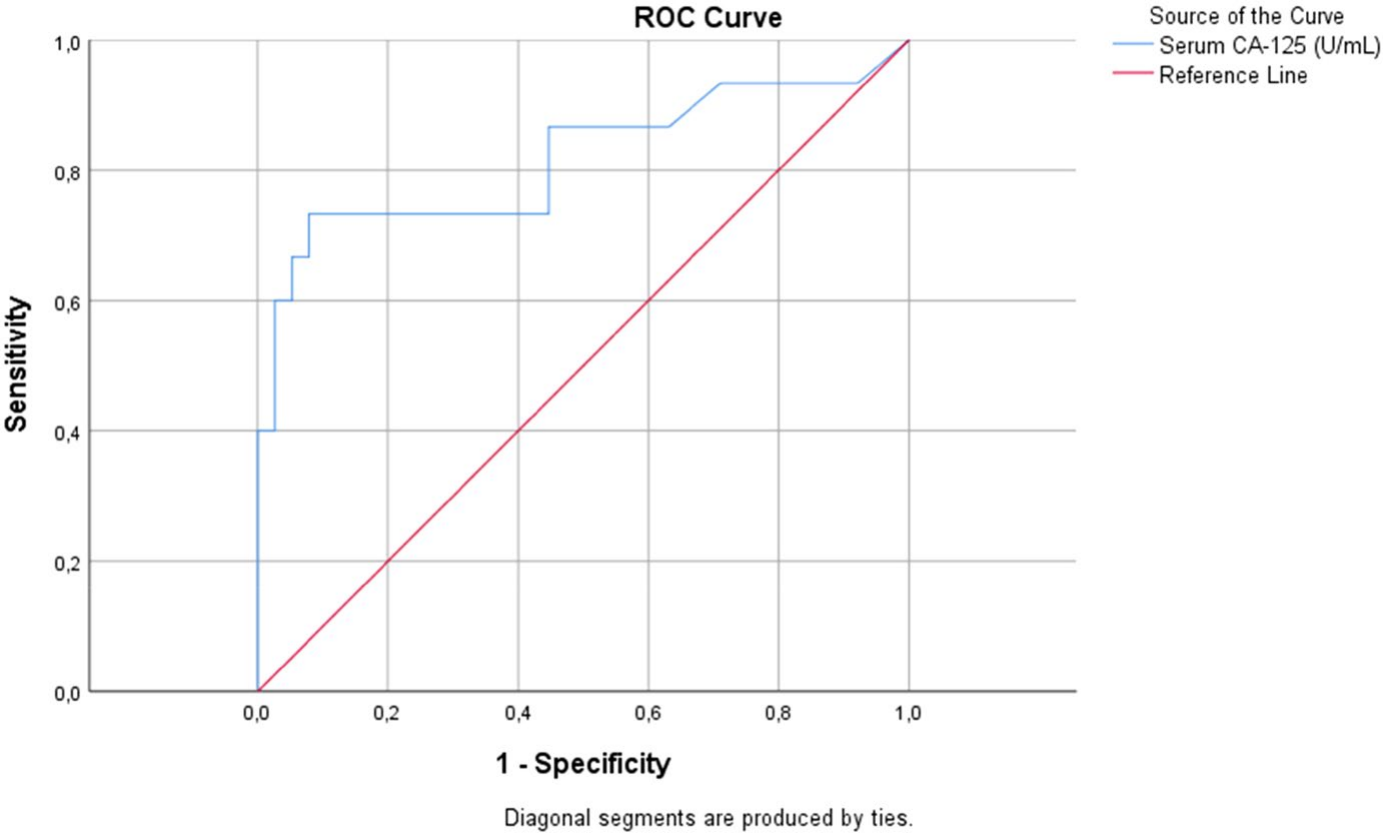
Receiver operating characteristic (ROC) curve analysis of serum cancer antigen 125 (CA125) levels for discriminating adipose‐ and muscle‐layer cesarean section scar endometriosis (area under the curve = 0.82, standard error = 0.08, *P* < 0.001).

The groups (adipose‐layer CSSE and muscle‐layer CSSE) differed significantly in terms of major symptoms, CSSE location, and preoperative serum CA125 levels (Table 1). Although the major symptom was swelling in the adipose‐layer CSSE group, pain was observed in the muscle‐layer CSSE group (27/38 [71.1%] vs. 12/15 [80.0%]; *P* = 0.001). Adipose‐layer CSSE were common at the corners; however, muscle‐layer CSSE were common at the midline (34/38 [89.5%] vs. 7/8 [87.5%]; *P* = 0.007). The muscle‐layer CSSE group had significantly higher mean preoperative serum CA125 levels than the adipose‐layer CSSE group (38.2 ± 19.2 U/mL vs. 18.5 ± 7.3 U/mL; *P* = 0.001). Receiver operating characteristic curve analysis revealed that the optimum cut‐off serum CA125 level for discriminating between the two groups was 30 U/mL, with a sensitivity and specificity of 75.0% (Figure 2). Binary logistic regression analysis revealed that the major symptom (i.e. pain) (OR 7.24; *P* = 0.031) and elevated serum CA125 levels (≥30 U/mL) (OR 19.63; *P* = 0.003) were independently associated with muscle‐layer CSSE (Table 2).

**TABLE 1:** Demographic and clinical characteristics of the adipose‐ and muscle‐layer cesareans section scar endometriosis groups.^a^

Characteristics	Adipose‐layer CSSE (*n* = 38)	Muscle‐layer CSSE (*n* = 15)	*P* value^b^
Age, years	32.9 ± 5.4	34.9 ± 6.8	0.267
Parity	2.1 ± 0.8	2.0 ± 1.0	0.842
No. of cesarean sections			
One	18 (34)	8 (15.1)	0.766
Two or more	20 (37.7)	7 (13.2)	
Time interval, months	39.8 ± 22.2	41.9 ± 27.2	0.525
Major symptom			
Pain	11 (20.8)	12 (22.6)	0.001
Swelling	27 (50.9)	3 (5.7)	
CSSE location			
Corners	34 (64.2)	8 (15.1)	0.007
Midline	4 (7.5)	7 (13.2)	
Serum CA125 level, U/mL	18.5 ± 7.3	38.2 ± 19.2	0.001

Abbreviations: CA125, cancer antigen 125; CSSE, cesarean section scar endometriosis.

^a^ Data are presented as mean ± standard deviation or as number (percentage).

^b^ Independent sample *t* tests and *χ* ^2^ tests were used to compare groups.

**TABLE 2:** Binary logistic regression analysis of factors associated with muscle‐layer cesarean section scar endometriosis.

Factor	*P* value	95% CI	OR
Main symptom (i.e. pain)	0.031	1.12–46.09	7.24
Elevated serum CA125 levels (≥30.0 U/mL)	0.003	3.03–126.83	19.63
Location of CSSE (i.e. at the midline of the cesarean section scar line)	0.440	0.28–17.82	—

Abbreviations: CA125, cancer antigen 125; CI, confidence interval; CSSE, cesarean section scar endometriosis; OR, odds ratio.

## 4 DISCUSSION

Cesarean section is one of the most commonly performed surgical procedures among women, and its incidence has steadily increased since the 1990s.^15^ Currently, approximately one in five women worldwide gives birth via cesarean section, and by 2030, one in three women is expected to give birth by cesarean section.^15^ Consequently, the incidence of CSSE will increase and a greater number of women are expected to experience this adverse condition. Additional research and clinical experience regarding this uncommon condition are therefore essential to improve the management of CSSE. This study revealed that the triad of symptoms, clinical suspicion, and abdominal ultrasonography had 92.9% accuracy in diagnosing CSSE. Additionally, although adipose‐ and muscle‐layer CSSE significantly differed in terms of major symptoms (i.e. pain or swelling), CSSE location (i.e. corners or midline), and preoperative serum CA125 levels, only the symptoms (i.e. pain) and elevated serum CA125 levels (≥30 U/mL) were independently associated with muscle‐layer CSSE.

The identification and accurate diagnosis of CSSE can be challenging because of its rarity.^3^ Diagnosis can be particularly challenging in patients without swelling or a palpable mass during physical examination, or when they consult specialists with limited expertise in this field.^10^, ^16^ Additionally, the differential diagnosis of CSSE is extensive, and includes benign and malignant neoplasms, abscesses, cysts, and chronic granulomatous reactions.^4^ Ultrasonography is commonly used as an initial diagnostic imaging tool because of its high accuracy and low cost.^9^, ^17^ Further diagnostic imaging tools, such as computed tomography and magnetic resonance imaging, can aid in the diagnosis.^6^, ^17^ Previous studies have reported wide variability in the accuracy of preoperative CSSE diagnoses, ranging from 45.0% to 97.4%.^9^, ^10^, ^14^, ^18^ In this study, the preoperative diagnostic accuracy was 92.9%, supported by a triad of symptoms, clinical suspicion, and abdominal ultrasonography.

Patients with CSSE have similar demographic characteristics and are typically parous women of reproductive age.^18^ In this study, all patients with CSSE were of reproductive age, with a mean age of 33.4 years and parity of 2.0. Similarly, previous studies involving a larger number of patients with CSSE indicated that the patients' mean age varied between 29.8 and 36.2 years and the mean parity varied between 1 and 2.^3^, ^4^, ^5^, ^7^, ^8^, ^9^, ^14^, ^18^ The relationship between sex hormones and endometriosis is well‐known and involves multiple mechanisms, including the epigenetic regulation of sex hormone receptors and interactions with the local inflammatory response.^2^ Hence, CSSE occurs in women of reproductive age, as observed in pelvic endometriosis. In this study, more than half of the patients had undergone two or more previous cesarean sections. In contrast to our findings, Zhang et al.^3^ and Hocaoglu et al.^14^ found that more than 80% of CSSE developed after the first cesarean section. However, insufficient data are available to determine the role of parity and the number of previous cesarean sections in CSSE. Furthermore, this study revealed no significant differences between the adipose‐ and muscle‐layer CSSE groups in terms of demographics, such as age, parity, and number of previous cesarean sections.

The exact etiology of CSSE remains unknown. The “iatrogenic direct implantation theory” is the most plausible explanation for this condition, suggesting that during a cesarean section, endometrial cells are implanted in the abdominal wound.^3^, ^4^ Although some authors suggest various strategies to prevent CSSE, such as focusing on surgical technique, using different sutures for closing the endometrial layer and fascia, and rinsing the wound with saline before abdominal closure, no data proving the effectiveness of these recommendations are available.^4^ These lesions require time for growth and to cause symptoms. In the present study, the mean time from the last cesarean section to the symptom onset was 40.4 months. Pain and swelling were the major symptoms, with a mean lesion diameter of 25.0 mm. Pelvic endometriosis was not detected on transvaginal ultrasonography in any of the cases, and CSSE was located within the adipose‐layer and at the corners of the cesarean section scar line in almost 75% of the cases. Previous studies have shown that patients with CSSE have clinical characteristics similar to those observed in the present study.^3^, ^4^, ^5^, ^6^, ^9^, ^14^ Previous studies have reported that the mean time to the onset of symptoms after the last cesarean section was between 28 and 44 months, with pain and swelling being the major symptoms, and the mean lesion size varied between 21 mm and 47 mm.^3^, ^4^, ^5^, ^9^, ^14^ The incidence of concomitant pelvic endometriosis was rare in patients with CSSE, ranging from 0% to 18%.^6^, ^11^, ^14^ Additionally, the most common positions of CSSE were the corners of the cesarean scar line and within the adipose‐layer of the abdominal wall.^3^, ^5^, ^9^, ^14^ However, this study showed that the adipose‐ and muscle‐layer CSSE groups differed significantly in terms of symptoms and CSSE location. Although adipose‐layer CSSE was commonly found at the corners, with swelling as the major symptom, muscle‐layer CSSE was more common at the midline, where pain was the predominant symptom. However, only pain was independently associated with muscle‐layer CSSE (OR 7.24). Zhong et al.^13^ reported that severe abdominal pain is a significant risk factor for postoperative recurrence. Further studies are needed to elucidate the relationship between pain, CSSE depth, and the risk of recurrence.

CA125 is a glycoprotein biomarker, and its serum levels have been extensively studied for the detection and monitoring of pelvic endometriosis.^19^ Elevated serum CA125 levels have demonstrated a statistically significant association with the severity of clinicopathologic parameters, including disease stage, adhesion score, and lesion size.^19^ However, the role of serum CA125 level in CSSE remains unclear. Only a few studies have examined serum CA125 levels in a small number of patients with AWE, and these values were within the normal range for most patients.^5^, ^7^ Therefore, some authors have concluded that there is no point in examining serum CA125 levels in either AWE or CSSE.^5^, ^7^, ^10^ In this study, the mean preoperative serum CA125 level was 24.1 U/mL, and only 20.7% of patients had serum CA125 levels of 35 U/mL or greater. Similarly, Ding and Zhu^5^ and Zhao et al.^7^ reported serum CA125 levels of at least 35 U/mL in less than 35.1% of patients with AWE. However, this study revealed that serum CA125 levels were significantly higher in patients with muscle‐layer CSSE, and the optimum cut‐off serum CA125 level for discriminating between the groups was 30 U/mL. Furthermore, elevated serum CA125 levels (≥30 U/mL) were independently associated with muscle‐layer CSSE (OR 19.63). Similar findings were also obtained for pelvic endometriosis, and serum CA125 levels greater than 30 U/mL were found to be higher in patients with both deep‐seated and severe endometriosis.^20^, ^21^ This may be related to enlarged or deeper‐seated lesions being closer to the lymphatics and circulation.^22^, ^23^

In summary, this study evaluated the clinical characteristics of patients with CSSE from a novel perspective, by focusing on the depth of placement within the abdominal wall. Our findings indicate that the major symptom (i.e. pain) and elevated serum CA125 levels (≥30 U/mL) were significantly associated with muscle‐layer CSSE. These findings may have important implications in clinical practice, including the potential localization of undefined lesions, surgical planning, and follow up of the risk of recurrence. This study has some limitations. First, the relatively small sample size; second, the retrospective design; third, selection bias because of the exclusion of certain patient groups; fourth, determining the presence of pelvic endometriosis through ultrasonography; and finally, the lack of long‐term follow up. Further research is needed to determine the clinical differences among the different layers of abdominal wall CSSE and to evaluate the role of serum cancer antigens in both diagnosis and follow up. Considering the rarity of CSSE, collaborative, multicenter, prospective studies could provide valuable insights into their nature and underlying mechanisms.

## AUTHOR CONTRIBUTIONS

EET conceived and designed the study; MIH acquired the data; EET, MIH, MD, and AFY analyzed and interpreted the data; EET, MIH, MD, and AFY drafted the manuscript. All authors read and approved the final manuscript and all authors meet ICMJE authorship criteria.

## CONFLICT OF INTEREST STATEMENT

The authors have no conflicts of interest.

## Supporting information


Data S1.

## Data Availability

Data sharing is not applicable to this article as no new data were created or analyzed in this study.
